# Mechanisms of Nucleosome Reorganization by PARP1

**DOI:** 10.3390/ijms222212127

**Published:** 2021-11-09

**Authors:** Natalya V. Maluchenko, Dmitry K. Nilov, Sergey V. Pushkarev, Elena Y. Kotova, Nadezhda S. Gerasimova, Mikhail P. Kirpichnikov, Marie-France Langelier, John M. Pascal, Md. Sohail Akhtar, Alexey V. Feofanov, Vasily M. Studitsky

**Affiliations:** 1 Faculty of Biology, Lomonosov Moscow State University, 119234 Moscow, Russia; maluchenko@gmail.com (N.V.M.); shordome@gmail.com (N.S.G.); kirpichnikov@inbox.ru (M.P.K.); 2 Belozersky Institute of Physicochemical Biology, Lomonosov Moscow State University, 119991 Moscow, Russia; nilovdm@gmail.com; 3 Faculty of Bioengineering and Bioinformatics, Lomonosov Moscow State University, 119991 Moscow, Russia; spush@fbb.msu.ru; 4 Fox Chase Cancer Center, Philadelphia, PA 19111-2497, USA; Elena.Kotova@fccc.edu; 5 Department of Biochemistry and Molecular Medicine, Université de Montréal, 2900 Boulevard Edouard-Montpetit, Montreal, QC H3T 1J4, Canada; mflangelier@mail.ru (M.-F.L.); john.pascal@umontreal.ca (J.M.P.); 6 Molecular and Structural Biology Division, CSIR-Central Drug Research Institute, Lucknow 226031, Uttar Pradesh, India; sohail@cdri.res.in; 7 Shemyakin-Ovchinnikov Institute of Bioorganic Chemistry, Russian Academy of Sciences, 117997 Moscow, Russia

**Keywords:** nucleosome, poly(ADP-ribose) polymerase 1, spFRET microscopy, molecular dynamics

## Abstract

Poly(ADP-ribose) polymerase 1 (PARP1) is an enzyme involved in DNA repair, chromatin organization and transcription. During transcription initiation, PARP1 interacts with gene promoters where it binds to nucleosomes, replaces linker histone H1 and participates in gene regulation. However, the mechanisms of PARP1-nucleosome interaction remain unknown. Here, using spFRET microscopy, molecular dynamics and biochemical approaches we identified several different PARP1-nucleosome complexes and two types of PARP1 binding to mononucleosomes: at DNA ends and end-independent. Two or three molecules of PARP1 can bind to a nucleosome depending on the presence of linker DNA and can induce reorganization of the entire nucleosome that is independent of catalytic activity of PARP1. Nucleosome reorganization depends upon binding of PARP1 to nucleosomal DNA, likely near the binding site of linker histone H1. The data suggest that PARP1 can induce the formation of an alternative nucleosome state that is likely involved in gene regulation and DNA repair.

## 1. Introduction

PARP1 is an abundant nuclear enzyme (1–2 million molecules per cell) and a nucleosome-binding protein localized in cell nuclei and involved in a variety of cellular processes including DNA repair, chromatin organization and transcription [1,2,3,4].

PARP1 has distinct catalytic activity-dependent and independent functions during DNA transactions [5,6]. There are two types of catalytic activity-dependent functions of PARP1. First, upon binding to DNA lesions, PARP1 becomes catalytically active, thus acting as a DNA damage sensor enzyme, which hydrolyzes NAD^+^ and forms long and branched chains of negatively charged polyADP-ribose (PAR) on a variety of nuclear proteins, including histones and PARP1 itself [7]. In this case, an interaction between the DNA-binding domain of PARP1 and DNA ends results inactivation of PARP1 [8,9,10].

This “hit and run” activation of PARP1 initiates the DNA repair pathway in cells [11]. The second catalytic activity-dependent function of PARP1 is mediated by interaction between the C-terminal domain of PARP1 and core histone H4 [11]. This binding results in long-term activation of PARP1 and continuous accumulation of PAR, which maintains chromatin in the loosened state around certain loci to guarantee that the transcription machinery has continuous access to DNA. PARP1 is responsible for 75–90% of the global PAR synthesis following DNA strand breakage [12,13,14]. Enzymatically active PARP1 could PARylate a number of regulatory proteins (e.g., KDM5B, transcription factors NELF, CTCF, AP-1, YY1, NF-kB, E2F-1, histones H1, H2A and H2B, p53, FACT and other proteins) involved in numerous cellular processes (reviewed in [2,15,16,17]).

The catalytic activity-independent function is realized during an interaction of PARP1 with intractable chromatin regions where it makes protein-binding sites on DNA available for interactions with pioneer factors. Thus, PARP1 facilitates binding of pioneer transcription factor Sox2 to intractable genomic loci and helps to maintain pluripotency in embryonic stem cells [5].

Finally, as a transcriptional regulator, PARP1 can interact with gene promoters where it likely binds to nucleosomes and replaces linker histone H1 [18,19]; in this case, it is unknown whether catalytic activity of PARP1 is required for gene regulation. 

The catalytic activity-independent function of PARP1 has been recapitulated in vitro: PARP1 facilitated Sox2 binding to Sox motifs hidden in nucleosomal DNA [5]. PARP1-dependent binding of Sox2 to the nucleosomes requires the DNA-binding domain of PARP1, which allows its binding to nucleosomal DNA and the PARP1 BRCT domain, which promotes interaction between PARP1 and Sox2 [5]. A similar catalytic activity-independent binding of PARP1 to nucleosomes has been demonstrated in vitro; it has been shown that this type of binding does not occur through DNA ends [20]. Furthermore, we have shown that during catalytic activity-independent binding of PARP1 to nucleosomes containing double-strand DNA breaks (DSBs) the structure of nucleosomes is considerably changed (reorganized) in a NAD+-independent way [21]. However, it remains unknown how PARP1 binding affects the structure of the nucleosome.

In the current study, we have shown that catalytic activity-independent binding of two or three PARP1 molecules to a core nucleosome or a nucleosome with a linker, respectively, is required to induce complete nucleosome reorganization in vitro. Binding of the third molecule of PARP1 and nucleosome reorganization require the presence of a 20 bp DNA region adjacent to the core nucleosome, suggesting that the binding occurs at the nucleosomal DNA exit/entrance region near the H1 binding site.

## 2. Results

### 2.1. Experimental Approaches for Analysis of PARP1-Dependent Changes in Nucleosome Structure

**Figure 1:**
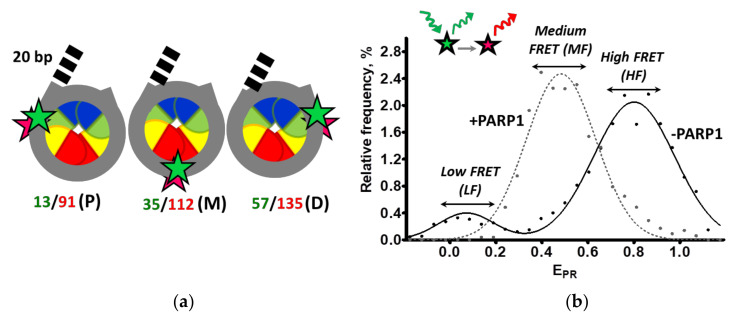
Experimental approach for analysis of PARP1-dependent changes in nucleosome structure using spFRET microscopy. (**a**) Positions of Cy3 and Cy5 fluorophores (green and red asterisks, respectively) on core 603-42A nucleosomes (CN) and nucleosomes containing an additional 20 bp DNA region (dashed lines, “linker”-containing nucleosomes, LN). Positions of Cy3 and Cy5 labels relative to the boundary of the 147 bp 603-42A nucleosome positioning DNA sequence (13/91, 35/112 and 57/135) are indicated using “bp” units. Depending on the label positions CN and LN nucleosomes are hereinafter referred as P-, M- and D-labeled. Histones are shown as colored semi-ovals: H2A—yellow, H2B—red, H3—blue, H4—green. (**b**) A schematic diagram of spFRET microscopy data analysis. Typical profiles of nucleosomes (−PARP1) and PARP1-nucleosome complex (+PARP1) are shown (approximated by the Gaussians); the typical peaks are shown.

Uniquely positioned nucleosomes were reconstituted on 603-42A DNA templates in vitro [22]. To make the nucleosomes suitable for analysis by single-particle Förster resonance energy transfer (spFRET) microscopy, a single pair of fluorescent dyes, Cy3 and Cy5, was introduced in different regions of the nucleosomal DNA (Figure 1a). Using this approach, we have shown previously that PARP1 can introduce structural changes (reorganize) in the DNA of nucleosomes containing a double-stranded break exposed at the end of a 20 bp DNA region (a linker) adjacent to the core nucleosome [21]. However, it remained unclear whether the observed reorganization of the nucleosome structure was caused by the interaction of PARP1 with the DNA end(s) (double strand breaks [8,9,10]) or with intact nucleosomal DNA [2,18,23]. To discriminate between these possibilities, both core- and single linker-containing nucleosomes were constructed (Figure 1a). In both cases Cy3/Cy5 were attached to the neighboring DNA gyres at positions 13/91 bp (P templates), 35/112 bp (M templates) or 57/135 bp (D templates) from the boundary of the 147 bp 603-42A nucleosome positioning DNA sequence (Figure 1a). These positions of the label pairs provide efficient FRET between the donor (Cy3) and acceptor (Cy5) fluorophores without disturbance of histone–DNA interactions in the assembled nucleosomes [21,24]. The labels at the positions 13 and 135 bp are localized in the regions of nucleosomal DNA involved in “breathing” [25]. The 35 and 112 bp labels are localized near the boundaries between DNA-bound H2A/H2B dimers and H3/H4 tetramers, whereas 57 and 91 bp labels are within the DNA regions interacting with the H3/H4 histone tetramer (Figure 1a).

Structural alterations in nucleosomes induced by PARP1 binding were studied using primarily spFRET microscopy in solution, which reveals changes in distances between labels (i.e., any changes in DNA folding on the surface of the histone octamer). The calculated frequency distributions of nucleosomes by E_PR_ values were used for the analysis of nucleosomal structural features (Figure 1b) [21,24]. E_PR_ is FRET efficiency without correction for quantum yields and detection sensitivity of fluorophores [26]. Typically, two peaks are observed in the E_PR_ profiles of nucleosomes: the high-FRET (HF) peak, which corresponds to intact nucleosomes, and the low-FRET (LF) peak reflecting presence of nucleosomes with unwrapped and/or histone-free DNA [21]. E_PR_ of an intermediate value (MF, Figure 1b) is typically observed in the presence of PARP1, indicating that there is a moderate increase in the spacing between the labels as compared with intact nucleosome. The formation of nucleosome–PARP1 complexes was also monitored by EMSA and accompanied by analysis of stoichiometry of separated complexes using single particle fluorescence intensity analysis of nucleosomes in gel. DNAseI footprinting was used to reveal regions of PARP1 binding on nucleosomal DNA. Finally, PARylation of the nucleosome–PARP1 complexes in the presence of NAD+ was studied using anti-PAR antibodies.

### 2.2. Characterization of the Nucleosomes and PARP1–Nucleosome Complexes

**Figure 2:**
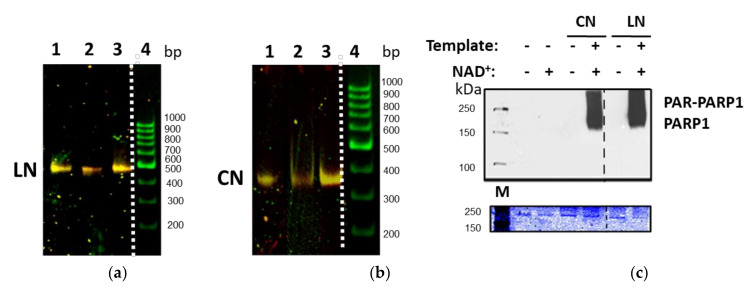
Characterization of the templates and PARP1–nucleosome complexes. (**a**,**b**) Fluorescently labeled purified nucleosomes were separated by non-denaturing PAGE: a. 1—LN_P, 2—LN_M, 3—LN_D, 4—nucleosomal 167 bp DNA. b. 1—CN_P, 2—CN_M, 3—CN_D. Presented images (**a**,**b**) were obtained by merging the images of Cy3 and Cy5 fluorescence distribution recorded at the excitation of Cy3 fluorophore. A color of bands corresponds to FRET efficiency, which increases in the following order: green < yellow < orange. (**c**) Catalytic activity of PARP1 is activated by core and linker-containing nucleosomes. PARP1 was incubated with CN or LN and/or NAD+, and proteins were analyzed by SDS-PAGE followed by Coomassie staining (bottom gel) or Western blot with anti-PAR-antibodies staining (top gel). M—protein markers.

Analysis of fluorescently labeled purified nucleosomes by non-denaturing PAGE indicates that they have characteristic mobilities, form single bands in the gel and are characterized by a higher FRET efficiency between the fluorophores than purified nucleosomal DNA (yellow vs. green color of the bands in the gel, respectively) (Figure 2a,b).

**Figure 3:**
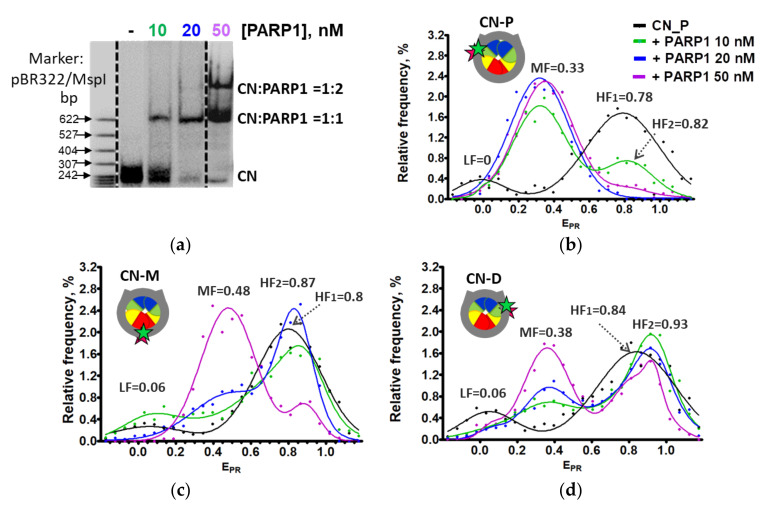

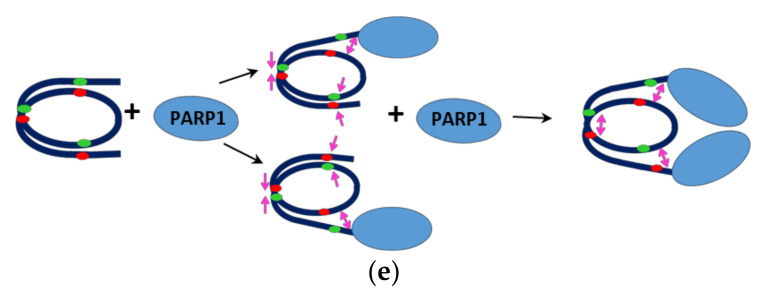
PARP1 forms structurally distinct complexes with core nucleosomes. (**a**) Analysis of the complexes by non-denaturing PAGE. (**b**–**d**) Analysis of PARP1 complexes with CN-P (**b**), CN-M (**c**) and CN-D (**d**) using spFRET microscopy**.** Typical E_PR_ distributions of nucleosomes are shown (number of experiments n = 3, N~3000 particles in each experiment). (**e**) Schematic diagram of different complexes formed between PARP1 and core nucleosomes. The changes in the distances between neighboring DNA gyres at the labeled sites as compared with intact nucleosomes are shown by arrows.

**Figure 4:**
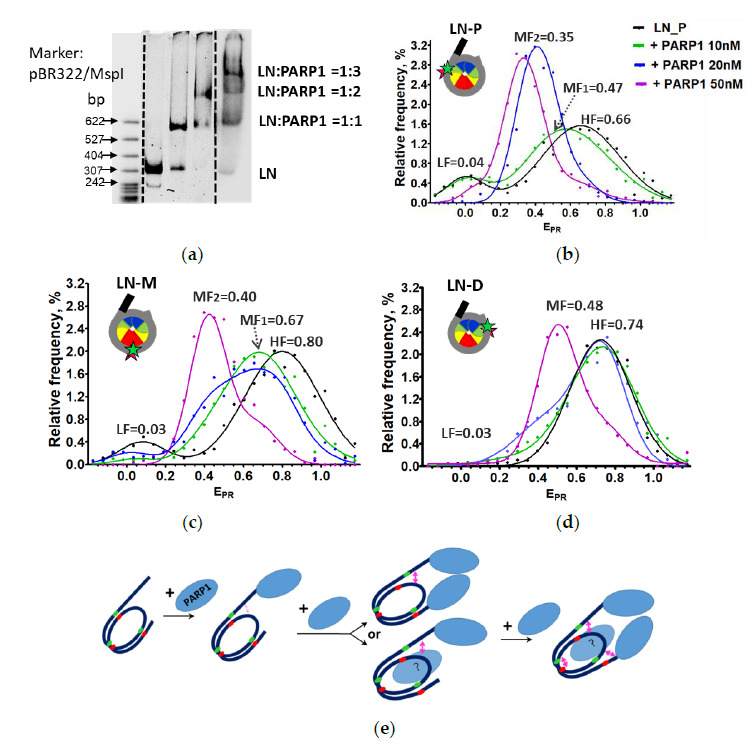
Nucleosomes containing an extended DNA region are fully reorganized only after binding of three molecules of PARP1. (**a**) Analysis of the PARP1–nucleosome complexes by non-denaturing PAGE. (**b**–**d**) Analysis of PARP1 complexes with LN-P (**b**), LN-M (**c**) and LN-D (**d**) using spFRET microscopy. Typical E_PR_ distributions of nucleosomes are shown (number of experiments n = 3, N~3000 particles in each experiment). (**e**) A schematic diagram of two possible variants of complex formation between PARP1 and a nucleosome. Other designations as in Figure 3. Curves (**b**–**d**): black—nucleosomes, green—added 10 nM PARP1, blue—added 20 nM PARP1, lilac—added 50 nM PARP1.

The structures of the nucleosomes and PARP1-nucleosome complexes were studied using DNase I footprinting (Figure S1). The complexes were treated with DNase I at two different PARP1 concentrations allowing the formation of either 1:1 or 1:2 PARP1–nucleosome complexes (see Figure 3a and Figure 4a). CN and LN are characterized by strong protection of nucleosomal DNA from the enzyme and by patterns of bands in a denaturing gel having ~10 bp periodicity that reflects periodic cutting of DNA regions exposed into solution on the surface of the histone octamer (Figure S1). No significant changes in the DNase I sensitivity patterns (i.e., changes in band intensities) in the nucleosome core upon binding of PARP1 to the nucleosomes were observed (Figure S1), indicating that DNA–histone contacts are not strongly disturbed in the PARP1–nucleosome complexes. A weak protection of the linker DNA of LN nucleosome in the presence of PARP1 was detected, consistent with a similar effect described by others previously [2,27].

Activity of PARP1 in the PARP1–nucleosome complexes was evaluated (Figure 2c). As expected, PARP1 is activated and autoPARylation of PARP1 occurs only in the presence of nucleosomes and NAD+.

### 2.3. PARP1 Forms Structurally Distinct Complexes with Core Nucleosomes

Analysis of PARP1-CN interaction using EMSA shows that a single additional, slowly migrating band, likely corresponding to PARP1–CN complexes is observed in the gel at lower PARP1 concentrations (10 and 20 nM), while another, even slower migrating band appears in the gel at a higher PARP1 concentration (50 nM), likely reflecting a change in stoichiometry of PARP1–CN complexes (Figure 3a).

To evaluate whether two (or more) nucleosomes are involved in the formation of the observed complexes, fluorescence intensities of single particles of both PARP1–CN complexes separated in gel were measured and compared with fluorescence intensities of single nucleosomes. Fluorescence intensities of single CN and single complexes of both types are similar (Supplementary Figure S2a), suggesting that both types of PARP1–nucleosome complexes contain one nucleosome per complex, and differences in their electrophoretic mobility are most likely related to different numbers of PARP1 molecules bound to the nucleosome. Thus, the data suggest that PARP1 forms complexes with CN having stoichiometry 1:1 (at 10 and 20 nM of PARP1) and 2:1 (at 50 nM of PARP1).

Analysis of frequency distributions of nucleosomes by E_PR_ shows that core nucleosomes are characterized by the presence of two subpopulations of nucleosomes: a major subpopulation with high E_PR_ values (high-FRET_1_ peak, HF_1_) and a minor subpopulation with low E_PR_ values (low-FRET peak, LF). The HF_1_ peak corresponds to intact nucleosomes, while the LF peak reflects the presence of nucleosomes with partially unwrapped DNA and/or free DNA. The formation of complexes between CN and increasing concentrations of PARP1 results in the disappearance of the LF peak and the gradual appearance of a new high-FRET_2_ peak (HF_2_, shifted and narrowed relative to the HF_1_ peak) and new medium-FRET (MF) peak (Figure 3b–d, Supplementary Figures S3 and S4a–c). The HF_2_ peak was detected at low PARP1 concentration, indicating the formation of complexes, in which distances between the neighboring DNA gyres become shorter in the regions of 13/91, 35/112 and 57/135 bp (since the value of E_PR_ peak maximum becomes higher) than in intact nucleosomes, and mobility of DNA is more restricted (since the HF_2_ peak is narrower than the HF_1_ peak). Probably, this restriction of the DNA mobility also results in the disappearance of the minor LF fraction of nucleosomes containing partially unwrapped DNA. The appearance of the MF peak indicates the formation of PARP1–nucleosome complexes, in which the distance between neighboring DNA gyres is increased. Thus, binding of one molecule of PARP1 to CN results in the formation of MF and HF_2_ nucleosomes at the same time. This can be explained by proposing that interaction of PARP1 with one end of 147 bp nucleosomal DNA results in an increase in the distance between DNA gyres near the binding region and stabilization of the other end of nucleosomal DNA on the histone octamer.

The MF subpopulation of nucleosomes arises at 10 nM PARP1 and becomes predominant at higher PARP1 concentrations (Figure 3b–d and Supplementary Figure S4a–c). At the 13/91 bp region, the conformational transitions are completed at 20 nM PARP1 and result in a decrease in the E_PR_ peak maximum from 0.78 ± 0.03 to 0.33 ± 0.05 (Figure 3b). A higher concentration of PARP1 (50 nM) is required to finalize structural changes in the 35/112 and57/135 bp regions of the nucleosomes, which result in a shift of the FRET peak maximum from 0.80 ± 0.02 to 0.48 ± 0.06 and from 0.84 ± 0.02 to 0.38 ± 0.04, respectively (Figure 3c,d). The changes in the E_PR_ profiles induced by PARP1 binding to the nucleosomes (high-FRET_2_ peak emergence followed by middle-FRET peak appearance) likely indicate the formation of two types of PARP1–nucleosome complexes.

At the lower PARP1 concentrations E_PR_ changes are more pronounced in the 13/91 bp than in the 57/135 bp region, suggesting that the 13/91 region is preferentially restructured after initial PARP1 binding. Thus, two ends of the 147 bp nucleosomal DNA are not equivalent in the ability to bind PARP1 (Figure 3b–d). Ability of PARP1 to interact with the DNA end near a nucleosome likely depends on the amplitude of “nucleosome breathing” (spontaneous reversible uncoiling of DNA from the histone octamer). In turn, this amplitude can be modulated by the strength of DNA–histone interactions, which differ in the 13 bp and 135 bp regions in the case of the 603 nucleosome positioning sequence [22,28].

Taken together, our spFRET data suggest the following model of partial reorganization of core nucleosomes upon binding of PARP1 (Figure 3e). The formation of 1:1 PARP1–CN complexes results in changes in the nucleosome structure that include: (i) an increase in the distance between neighboring DNA gyres involving at least 13 bp from the end of nucleosomal DNA bound by PARP1; (ii) a decrease in the inter-gyre distance and restriction of the DNA mobility in the middle part of CN and at the other end of nucleosomal DNA (Figure 3e). The formation of the 2:1 PARP1–CN complexes induces considerable changes in the entire nucleosome structure—an increase in the distance between neighboring DNA gyres involving at least 35 bp from each end of the nucleosomal DNA (Figure 3e).

### 2.4. Nucleosomes Containing an Extended DNA Region Are Fully Reorganized Only after Binding of Three Molecules of PARP1

EMSA revealed the formation of three distinct PARP1–LN complexes (Figure 4a). The first PARP1–LN complex is observed at a low PARP1 concentration (10 nM). Second and third complexes having a lower mobility in the gel appear at 20 nM and 50 nM PARP1, respectively. To evaluate stoichiometry of the PARP1–LN complexes, fluorescence intensities of different single complexes separated in gel were compared (Supplementary Figure S1b). Frequency distributions of these intensities are similar, indicating that the three bands observed in the EMSA gel contain one nucleosome per complex and most likely correspond to the complexes of a nucleosome with 1, 2 and 3 molecules of PARP1.

Next, we extended our spFRET analysis of LN nucleosomes conducted previously [21] to a wider range of PARP1 concentrations, allowing the formation of different types of PARP1–LN complexes (Figure 4b–d, Supplementary Figures S4d–f and S5).

The calculated E_PR_ profiles of LN (Figure 4b–d) can be described as a superposition of two Gaussian peaks [21], where the HF peak corresponds to intact nucleosomes and the LF peak corresponds to nucleosomes with partially unwrapped DNA and/or histone-free DNA. The E_PR_ profiles of LN (LN-P and LN-M) in the presence of PARP1 (Figure 4b–d) are described by four Gaussian peaks: in addition to HF and LF peaks corresponding to LN, two additional middle FRET peaks (MF_1_ and MF2) were detected in the presence of PARP1 (Supplementary Figure S5). The E_PR_ profiles of PARP1–(LN-D) complexes are described by three Gaussian peaks (LF, MF and HF, Supplementary Figure S6). Contributions of MF peaks to E_PR_ profiles vary as a function of PARP1 concentration (Supplementary Figure S4d–f). At lower concentrations of PARP1, the HF peak decreases and the MF_1_ peak increases proportionally, having maxima at 0.47 ± 0.03 and 0.67 ± 0.04 for LN-P and LN-M, respectively (Figure 4b,c). At higher PARP1 concentrations, the MF_2_ peak appears and becomes predominant at 50 nM PARP1, with maxima at 0.35 ± 0.03, 0.40 ± 0.02 for LN-P and LN-M, respectively (Figure 4b,c). For LN_D nucleosomes, a considerable increase in the MF peak is observed at higher (50 nM) PARP1 concentration (Figure 4d). The data suggest that complete nucleosome reorganization occurs at the 13/91, 35/112 and 57/135 bp regions only at 50 nM PARP1, when three molecules of the protein are bound to the nucleosome (Figure 4e).

Combined EMSA and spFRET data suggest that the formation of the 1:1 PARP1–LN complex at 10 nM PARP1 is accompanied by minor changes in the distances between DNA gyres at the 13/91 and 35/112 bp regions (Figure 4b,c). At this concentration PARP1 most likely binds to the linker DNA end having a higher affinity to the protein. The formation of the 2:1 complex at 20 nM PARP1 is accompanied by more pronounced structural changes at the 13/91 bp DNA region (Figure 4b,e), resulting in the appearance of the pronounced MF_2_ peak in the E_PR_ profile. The second PARP1 molecule can bind to either the extending DNA end or nucleosomal DNA (Figure 4e); it is difficult to distinguish between these two binding modes. In contrast with CN nucleosomes where only two PARP1 molecules can bind at 50 nM PARP1 (Figure 3a), the third PARP1 molecule binds to the LN nucleosome at this concentration of PARP1, inducing additional structural changes at the 35/112 and 57/135 bp regions of nucleosomal DNA, reflected in the appearance of the dominating MF_2_or MF peaks in the E_PR_ profiles or LN-M or LN-D nucleosomes, respectively (Figure 4b–d). The exact location of the third binding site of PARP1 on the nucleosome is unknown; most likely PARP1 binds to the core region of the nucleosome, inducing a major structural reorganization of the entire nucleosomal DNA (see Section 3).

### 2.5. Interaction between Two PARP1 Molecules Bound to Double Strand DNA Breaks Can Induce Changes in the Structure of Nucleosomes: MD Simulations

To discriminate between alternative binding scenarios (Figure 4e), we performed molecular dynamics (MD) modeling of two PARP1–nucleosome complexes, where one PARP1 molecule is bound to the linker DNA end or two PARP1 molecules are bound to both DNA ends. Since the location of the third binding site of PARP1 on nucleosomal DNA is not known, modeling of the complex containing three PARP1 molecules bound to the nucleosome was not conducted.

**Figure 5:**
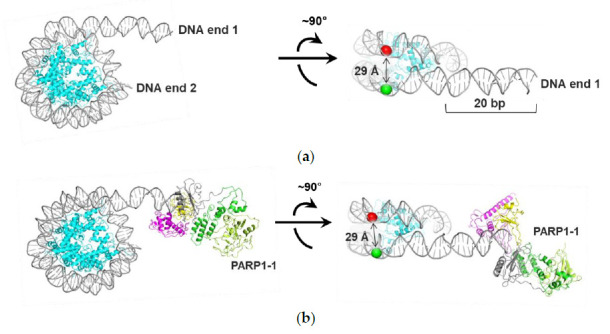

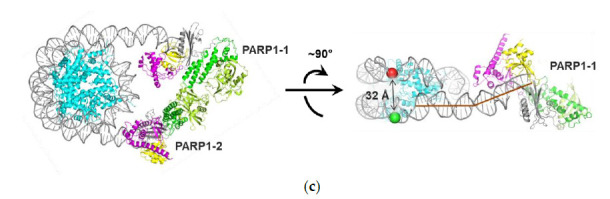
Interaction between two PARP1 molecules bound to double strand DNA breaks can induce changes in the structure of nucleosomes: MD simulations. The following complexes were studied using MD modeling: (**a**) A nucleosome; (**b**) complex of a nucleosome with one PARP1 molecule (PARP1-1); (**c**) complex of a nucleosome with two PARP1 molecules. Two orientations of the complexes are shown. Histones are shown in cyan; Zn1 domain of PARP1 in yellow, Zn3 domain in purple, WGR domain in gray. (ADP-ribosyl) transferase and helical subdomains of the PARP1 catalytic domain are shown in light and dark green, respectively. Circles indicate the positions of the C1′ atoms of the nucleotides positioned 13 (green) and 91 (red) bp from the nucleosome boundary proximal to the extended DNA region where the Cy3 and Cy5 labels were attached (see Figure 1a). PARP1 molecules bound to the linker and core DNA ends are dimerized during MD simulations (also see Supplementary Figure S7). The extended DNA region in model (**c**) is bent upon interaction between PARP1 molecules (compare with (**b**)); accordingly, the distance between the 13 and 91 nucleotides increases. The second PARP1 molecule is not shown on the right for clarity.

PARP1–nucleosome interactions were modeled using the 603-42A nucleosome with the 20 bp extended DNA region (linker). Three molecular models were constructed using available structures of a nucleosome [29] and PARP1–DNA complex with a double-stranded oligonucleotide [9]: (a) the nucleosome itself (Figure 5a), (b) the nucleosome containing one PARP1 molecule bound at the linker DNA end (Figure 5b), and (c) the nucleosome containing two PARP1 molecules bound at both linker and core DNA ends (Figure 5c). The ends of nucleosomal DNA served as a model of double strand DNA breaks.

For each starting model, an equilibration and subsequent 25 ns MD simulation were carried out. The duration of the equilibration stage was established by monitoring the distance between the C1′ atoms of the positioned nucleotides 13 and 91 (if we count from the beginning of the 603-42 DNA sequence) and by monitoring root-mean-square deviation (RMSD) of the nucleosome backbone (Supplementary Figures S8–S10). The equilibration stages were 1 ns long for model (a), 10 ns for model (b) and 15 ns for model (c).

The subsequent 25 ns equilibrium simulation revealed differences in the relative positions of the C1′ atoms of the nucleotides 13 and 91 (sites of attachment of the Cy3 and Cy5 labels) (Supplementary Figures S11 and S12). The distances between the C1′ atoms were 29.2 ± 1.3 Å in the nucleosome (Figure 5a), 29.1 ± 1.6 Å in the PARP1–nucleosome complex (Figure 5b), and 32.2 ± 1.4 Å in the 2xPARP1–nucleosome complex (Figure 5c). The monitoring of distances between C1′ atoms in middle or distal nucleosome regions (between nucleotides 35 and 112 or 57 and 135) did not reveal significant changes compared with the free nucleosome. The mean 35–112 nucleotide distance was ≈22 Å, and the mean 57–135 nucleotide distance was ≈29 Å in all MD models.

Analysis of the model of 2xPARP1 –nucleosome complex showed that two PARP1 molecules bound to the linker, and core DNA ends did not interact directly in the starting structure, but approached each other during the equilibration (Supplementary Figure S7) forming intermolecular interactions between the catalytic domains. This interaction, in turn, induced a distortion of linker DNA accompanied by an increase in the distance between the nucleotides 13 and 91 localized near the linker (Figure 5c). The observed conformational change resembles a pincer movement, with two PARP1 molecules acting as closing jaws.

Thus, according to the MD study, the binding of the first PARP1 molecule did not significantly affect the nucleosome structure, while the binding of the second PARP1 molecule induced reorganization of the region of nucleosomal DNA proximal to the linker DNA. 

Qualitatively, these MD data are in agreement with the spFRET microscopy data for LN at the PARP1 concentrations, which correspond to the binding of one or two PARP1 molecules to a nucleosome. Therefore, MD simulations suggest that PARP1 initially binds to both DNA ends, while the third PARP1 molecule interacts with nucleosomal DNA (Figure 4e).

Taken together, the data shown in Figure 3 and Figure 4 suggest that PARP1 can form several complexes with a nucleosome and induce structurally similar nucleosome reorganization involving the entire nucleosomal DNA by two ways: (a) through interaction of two molecules of PARP1 with two DNA breaks localized at the core nucleosome boundaries (Figure 3e); (b) through interaction of three PARP1 molecules with a nucleosome having two DNA breaks localized at the core nucleosome boundary and on an extended DNA linker (Figure 4e).

## 3. Discussion

The data suggest that upon binding of multiple molecules of PARP1 to core nucleosomes or nucleosomes containing a 20 bp linker DNA region (Figure 1a), the entire nucleosome structures are considerably reorganized (Figure 3 and Figure 4). The complete nucleosome reorganization requires two or three molecules of PARP1 interacting with core and linker-containing nucleosomes, respectively; however, in both cases structurally similar states of nucleosomes are achieved (Figure 3 and Figure 4). Sub-stoichiometric binding of PARP1 results in only local changes in the structure of nucleosomal DNA (Figure 3 and Figure 4). Full PARP1-induced nucleosome reorganization was detected using an experimental system where two ends of nucleosomal DNA model two closely situated DNA breaks in chromatin. Although the probability of such type of DNA damage in vivo is low, even PARP1 binding to a single dsDNA damage site situated near a nucleosome could induce partial nucleosome reorganization. Therefore, the observations of PARP1-induced nucleosome reorganization raise several important questions about its mechanism and role in a cell.

How does the binding of two PARP1 molecules induce the reorganization of CN? In this simpler case, each PARP1 molecule binds to and locally displaces one end of nucleosomal DNA from the surface of the histone octamer, and the combined action of two PARP1 molecules results in more extensive nucleosome reorganization (Figure 3e). This scenario explains why two PARP1 molecules do not fully reorganize the LN nucleosome having a 20 bp extended linker DNA: in this case, only one of two bound PARP1 molecules is positioned close to the core region and induces local reorganization of the nucleosome. Interaction between two PARP1 molecules bound at the ends of LN nucleosomal DNA facilitates this local reorganization but does not induce full nucleosome reorganization (Figure 4 and Figure 5).

**Figure 6:**
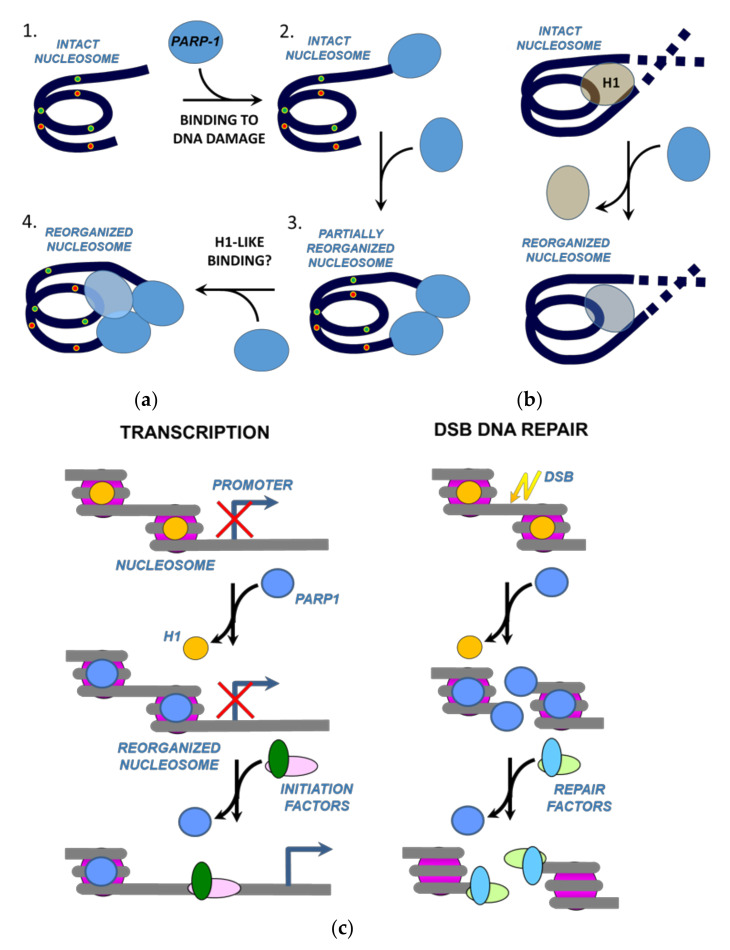
Possible roles of PARP1-reorganized nucleosomes. (**a**) PARP1 can interact with a nucleosome containing dsDNA breaks (complex 1) through binding to the damage at the extended DNA end (complex 2) and/or to the DNA end at the boundary of nucleosomal DNA (complex 3); this binding does not prevent binding of an additional PARP1 molecule and formation of putative H1-like complex (complex 4). (**b**) PARP1 can replace linker histone H1 and possibly form H1-like complexes and reorganize nucleosomes. (**c**) Possible roles of PARP1-reorganized nucleosomes. Role in transcription initiation (on the left): During gene activation, PARP1 is recruited to activated promoters where it replaces histone H1. PARP1-reorganized nucleosomes are more accessible to transcription initiation factors and chromatin remodeling enzymes that induce nucleosome displacement and facilitate transcription. Role in DNA DSB repair (on the right): As PARP1 recognizes DNA DSB, its concentration is locally increased at the damage site, resulting in its binding both to DNA ends and H1-like binding to nucleosomes. Both types of binding could induce nucleosome destabilization that in turn facilitates recruitment of DNA repair factors.

Why at the same PARP1 concentration do two PARP1 molecules bind to CN and three –to LN nucleosomes? Two non-exclusive possible explanations are: (a) steric restrictions on binding of the third molecule by two PARP1 molecules bound to DNA ends, and (b) the presence of DNA linker facilitating binding of the third molecule. Both possibilities indicate that PARP1 binds to a site on the nucleosome near the entrance and exit of nucleosomal DNA that is characteristic of binding of linker histone H1 (Figure 6a). A similar DNA DSB-independent binding of PARP1 to nucleosomes has been demonstrated previously in pioneering studies from Luger laboratory [20,30]; however, these studies did not address possible structural changes in nucleosomes upon PARP1 binding. Since two molecules of PARP1 do not induce full reorganization of LN nucleosomes, it is likely that H1-like binding of the third molecule of PARP1 itself induces the extensive structural changes in the nucleosome (Figure 6). One cannot exclude a possibility that two closely positioned PARP1 molecules bound to a nucleosome can interact. However, currently we have no experimental confirmation of such interaction, and its hypothetical functional role is not clear. It should be mentioned that PARP1–PARP1 interactions in the absence of nucleosomes were found only in crystals and were not observed in solution [31].

Taken together, the data suggest that very different interactions of PARP1 with nucleosomal DNA can induce a structurally similar reorganized state of a nucleosome (Figure 3 and Figure 4). The characteristic feature of this state is an increased distance between the adjacent DNA helices as compared with the intact nucleosome (Figure 3 and Figure 4). Since very different interactions can induce it, this is likely a relatively stable alternative nucleosome state that exists at a local energetic minimum.

The existence of the PARP1-reorganized nucleosome state could explain the role of PARP1 bound at promoters of active genes, where it displaces linker histone H1, promotes the formation of chromatin that is permissive to gene expression [18] and, therefore, probably contains destabilized nucleosomes. Accordingly, we propose that H1-like binding of PARP1 destabilizes promoter nucleosomes through displacement of linker histone H1 and nucleosome reorganization, possibly promoting binding of transcription initiation factors and RNA polymerase II and increasing the efficiency of transcription initiation (Figure 6c). This nucleosome-destabilizing mechanism could constitute a second pathway involving catalytically inactive PARP1 and possibly facilitating the interaction of various factors with nucleosomal DNA; the first mechanism involves cooperative binding of PARP1 and pioneer transcription factor Sox2 to nucleosomal DNA [5].

It is also possible that the putative H1-like binding of PARP1 to nucleosomes and nucleosome reorganization play a role in DNA repair. According to the current view, PARP1 is a sensor protein that binds to the damaged DNA through the N-terminally localized zinc finger domains. Binding to a DNA damage triggers global conformational changes of PARP1 that ultimately lead to activation of its enzymatic function [10]. Activated PARP1 drives PARylation of various proteins including histones and PARP1 itself, resulting in efficient DNA repair. In addition to this scenario, we propose that recruiting of PARP1 to a DNA damage could increase its local concentration to a level that is sufficient for H1-like binding and reorganization of nucleosomes containing the DNA damage by additional molecules of the protein (Figure 6c); DNA in reorganized nucleosomes is likely more accessible to DNA repair proteins. The PARP1–PARP1 interaction could also induce limited nucleosome reorganization after binding to properly positioned DSBs (Figure 5). Thus, PARP1 could facilitate DNA repair both through its enzymatic and non-enzymatic functions.

## 4. Materials and Methods

### 4.1. Purification of Proteins, DNA Templates and Nucleosomes

Human recombinant full-sized PARP1 was expressed in *E. coli* and purified as described [9]. Electrophoregram of purified PARP1 is presented in Supplementary Figure S13.

The 147 bp and 167 bp DNA templates containing pairs of fluorescent labels Cy3 and Cy5 at different positions were obtained by polymerase chain reaction (PCR) using the nucleosome-positioning sequence 603-42A [22]. For the 167 bp DNA templates, design of fluorescently labeled oligonucleotides was described previously [21]. To produce 147 bp DNA templates, the following fluorescently labeled primers (Lumiprobe, Moscow, Russia) were used (Table 1):

The PCR products were purified from 2% agarose gel and extracted using QIAquick Gel Extraction Kit (Qiagen) following the manufacturer’s protocol.

Nucleosomes were assembled using fluorescently labeled DNA templates and chicken donor chromatin without linker histone H1, as described in [32]. Assembled nucleosomes were analyzed by the electrophoresis in non-denaturing 4.5% polyacrylamide gel (acrylamide/bisacrylamide 39:1; 0.5× TBE buffer, pH 8.0). Assembled nucleosomes were isolated from the gel by extraction in a buffer containing 10 mM HEPES-NaOH (pH 8.0), 0.2 mM EDTA, 0.2 mg/mL bovine serum albumin and stored at 4 °C. The purified nucleosomes contained less than 3% of histone-free DNA (Figure 2a,b).

### 4.2. spFRET Experiments in Solution

spFRET experiments were performed in a buffer containing 20 mM Tris-HCl (pH 7.9), 5 mM MgCl_2_, 150 mM KCl. Fluorescently labeled nucleosomes (1 nM) were incubated in the presence of different concentrations of PARP1 in low-adhesion tubes for 30 min at 25 °C. All the spFRET measurements were performed when an equilibrium state in complex formation was achieved.

spFRET measurements on freely diffusing nucleosomes in solution were performed as described previously [21]. Each measured single nucleosome was characterized by FRET between Cy3 and Cy5 labels calculated as a proximity ratio (E_PR_):

E_PR_ = (I_5_ − 0.19 × I_3_)/(I_5_ + 0.81 × I_3_), (1)

where I_3_ and I_5_ are fluorescence intensities of Cy3 and Cy5, respectively, and coefficients 0.19 and 0.81 provide correction for the spectral cross-talk between Cy3 and Cy5 detection channels. E_PR_ is a FRET efficiency without correction for quantum yields of labels and an instrumentation factor. Relative frequency distributions of E_PR_ values (2000–5000 nucleosomes per experiment; 3 independent experiments) were plotted and further analyzed as a superposition of two (free nucleosomes), three or four (PARP1–nucleosome complexes) Gaussian peaks (Supplementary Figures S2, S4 and S5). Decision on the number of Gaussian peaks, which are required to describe the E_PR_ profiles, was made based on the calculation of RMSD for alternative variants of the description. All E_PR_ profiles obtained for a particular type of nucleosomes (CN_P, CN_M, CN_D, LN_P, LN_M or LN_D) were subjected to the RMSD analysis. Sub-fractions of nucleosomes corresponding to different conformational states were calculated as ratios of areas under particular Gaussian peaks to the total area under the experimental curve (in percentages, Supplementary Figure S3). Results of the analysis were averaged.

### 4.3. EMSA (Electrophoretic Mobility Shift Assay) Experiments

Nucleosomes (2–3 nM) were incubated (30 min, +25 °C) with PARP1 in the TB150 buffer (20 mM Tris-HCl (pH 7.9), 5 mM MgCl_2_, 150 mM KCl, 5% sucrose). After incubation, the probes were analyzed by electrophoresis in non-denaturing 4% polyacrylamide gel (PAGE, acrylamide/bisacrylamide 59:1; 0.2×TBE buffer). The gels were scanned using a Typhoon PhosphoImager. Fluorescence was excited in the gel at the 532 nm wavelength and recorded in the 570–610 nm (for Cy3) and 650–700 nm (for Cy5) spectral regions.

### 4.4. Single Particle Fluorescence Intensity Analysis of Nucleosomes in the Gel

For the analysis of stoichiometry of the PARP1–nucleosome complexes, EMSA gels containing separated complexes were fixed between object and cover glasses and subjected to single particle fluorescence intensity analysis with the setup described previously [21]. Fluorescent images of the gels obtained with Typhoon PhosphoImager were used for navigation, and positions of bands with nucleosomes and their complexes were found in gels by scanning. A region of the selected band, where concentration of nucleosomes (or their complexes) was low enough for single particle measurements, was subjected to the analysis. Measurements were performed using the 633 nm excitation wavelength and the 650–800 nm detection range, thus exciting and detecting fluorescence of Cy5 only. Recording of single particle signal intensities was accompanied by moving periodically (each 5–10 s) laser focus position along the band over 10 μm region. Each measured single nucleosome or single complex was characterized by I_5_, and relative frequency distributions of I_5_ values (2000–3000 particles per experiment) were compared for free nucleosomes and nucleosome–PARP1 complexes from different bands in the gel.

### 4.5. Western Blot (WB) Experiments

Nucleosomes were incubated with PARP1 and NAD^+^ for 40 min in the EMSA buffer and subjected to electrophoresis in 4–12% bis-Tris gradient gel in NuPAGE™ MESSDS Running Buffer (50 mM MES, 50 mM Tris, 0.1% SDS, 1 mM EDTA, pH 7.3; Thermo Fisher Scientific, Waltham, MA, USA) at 130 V. Protein transfer on PVDF membrane was performed in the transfer buffer (25 mM Bicine 25 mM Bis-Tris (free base) 1 mM EDTA pH 7.2; Thermo Fisher Scientific) with 20% methanol at 4 °C and 70 V for 1 h. The membrane was incubated for 60 min in the 1× PBS-T solution (37 mM NaCl, 2.7 mM KCl, 8 mM Na_2_HPO_4_, 2 mM KH_2_PO_4_, 0.5% Tween 20) supplemented with the 5% skimmed milk (prepared from dry powder) and washed with the 1× PBS-T solution for 5 min. Then the membrane was incubated with primary antibodies against non-catalytic part of PARP1 (α-PARP) or mouse monoclonal antibodies against poly-ADP-ribose (clone 10H, Tulip BioLabs, Montgomery County, PA, USA) for 60 min in 1× PBS-T/5% milk. Then the membrane was incubated with the secondary anti-mouse antibodies conjugated with HRP (Bio-Rad, Hercules, CA, USA) or streptavidin-HRP for 60 min in the 1× PBS-T/5% milk solution. The washing procedure was carried out after each step of incubation. Immunodetection was performed using SuperSignal West Pico Chemiluminescent Substrate (Thermo Fisher Scientific) for 3 min.

### 4.6. DNaseI Footprinting

Oligonucleotides (10 µM) were incubated (40 min, 37 °C) with ^32^P-γATP (Perkin Elmer, Waltham, MA, USA) and T4 PNK kinase (NEB, Ipswich, MA, USA) in PNK buffer (NEB, Ipswich, MA, USA). Enzyme was inactivated by increasing temperature to 60 °C for 20 min. The product (5′-labeled oligonucleotide) was then purified using IllustraMicroSpin G-25 microcolumns (GE Healthcare, Chicago, IL, USA) according to the manufacturer’s manual.

DNA or nucleosomes (30 ng) were incubated with PARP1 (50 or 100 nM) for 25 min at 25 °C in 50 µL of (buffer) containing 20 mM Tris-HCl (pH 7.9), 5 mM MgCl_2_, 40 mM KCl. Then CaCl_2_ (to 0.5 mM), bovine serum albumin (10 μg) and Dnase I (0.2 U) were added into the reaction tube for 20 s at 25 °C. The reaction was stopped by adding 1 μL of 0.5 M EDTA. After that, DNA was purified using phenol/chloroform extraction and ethanol precipitation. The samples were analyzed by denaturing PAGE. The gels were quantified using a PhosphorImager.

### 4.7. Molecular Modeling

Three distinct nucleosome models were constructed: (a) free nucleosome, (b) complex with one PARP1 molecule and (c) complex with two PARP1 molecules. Model (a) was built based on the 3lz0 crystal structure of the 601 nucleosome core particle [29]. The 601 DNA sequence was replaced with the 603-42A DNA sequence [29], and a linker DNA arm (20 bp) was added using the 3DNA software [33]. Model (b) was built by combining model (a) and the PARP1 structure 4dqy containing a double-stranded DNA oligonucleotide [9]. To obtain the coordinates of PARP1 bound to the linker DNA end, the oligonucleotide backbone of 4dqy was superimposed onto the 20 bp linker backbone. Model (c) was obtained by introducing a second PARP1 molecule into model (b). For this purpose, the core DNA end (20 bp) was unwrapped using the 3DNA software, and PARP1 was positioned as described for model (b). The coordinates of missing loops in the PARP1 structure were predicted in our previous work [34].

For each starting model, an equilibration and subsequent 25 ns MD simulation were carried out using the Amber20 package [35]. Hydrogen atoms were added to the protein/DNA structures, and then they were solvated by a 12 Å thick layer of TIP3P water. Sodium ions were added to neutralize the negative net charge. The obtained systems were energy-minimized in two stages, one with positional restraints on the protein and DNA atoms (2500 steepest descent steps +2500 conjugate gradient steps) and the other without restraints (5000 steepest descent steps +5000 conjugate gradient steps). The minimized systems were heated up from 0 to 300 K with positional restraints (constant volume, 250 ps) and equilibrated at 300 K without restraints (constant pressure). The final stage was an equilibrium simulation over 25 ns, producing a trajectory for analysis. The integration step was 0.002 ps, periodic boundary conditions were applied. The *ff14SB* and *bsc1* force fields were used to describe the protein and DNA molecules [36,37].

## Figures and Tables

**Table 1:** Fluorescently labeled primers used to produce 147 bp DNA templates.

Name	Nucleotide Sequence
CN_P_forward	5′-CCCGGTTCGCGC[Cy3-dT]CCCGCCTTCCGTGTGTTGTCGTCTCTCGG-3′
CN_P_reverse	5′-ACCCCAGGGACTTGAAGTAATAAGGACGGAGGGCCTCTTTCAACATCGATGCACGG[Cy5-dT]GGTTAG-3′
CN_M_forward	5′-CCCGGTTCGCGCTCCCGCCTTCCGTGTGTTGTCG[Cy5-dT]CTCTCGG-3′
CN_M_reverse	5′-ACCCCAGGGACTTGAAGTAATAAGGACGGAGGGCC[Cy3-dT]CTTTCAACATCGAT-3′
CN_D_forward	5′-CCCGGTTCGCGCTCCCGCCTTCCGTGTGTTGTCGTCTCTCGGGCGTCTAAGTACGC[Cy3-dT]TAGGC-3′
CN_D_reverse	5’-ACCCCAGGGACT[Cy5-dT]GAAGTAATAAGGACGGAGGGCCTCTTTC-3′

## Data Availability

The data presented in this study are available on request from the corresponding author. The data are not publicly available due to local regulations.

## Supplementary Materials

The following materials are available online at https://www.mdpi.com/article/10.3390/ijms222212127/s1, Figure S1: PARP1 minimally affects DNA-histone interactions in the nucleosome, Figure S2: Analysis of stoichiometry of PARP1 complexes with core or linker nucleosomes using single particle fluorescence intensity analysis in gel, Figure S3: Typical examples of the description of Epr profiles of CN_P nucleosomes and their complexes with PARP1 as a superposition of several Gaussians, Figure S4: Analysis of spFRET data for complexes of PARP1 with CN (top) and LN (bottom) nucleosomes, Figure S5: Typical examples of the description of Epr profiles of LN_P nucleosomes and their complexes with PARP1 as a superposition of several Gaussians, Figure S6: Typical examples of the description of Epr profiles of LN_D nucleosomes and their complexes with PARP1 as a superposition of several Gaussians, Figure S7: Interaction of PARP1 molecules bound to the ends of nucleosomal DNA, Figure S8: MD equilibration of the free nucleosome model, Figure S9: MD equilibration of the model of nucleosome with one PARP1 molecule, Figure S10: MD equilibration of the model of nucleosome with two PARP1 molecules, Figure S11: Comparison of equilibrium MD simulations of the free nucleosome model and the model of nucleosome with one PARP1 molecule, Figure S12: Comparison of equilibrium MD simulations of the free nucleosome model and the model of nucleosome with two PARP1 molecules, Figure S13: 12% SDS-PAGE of purified PARP1.

## Abbreviations

PARP1	poly(ADP-ribose)polymerase 1
PAR	poly(ADP-ribose)
MD	molecular dynamics
FRET	Förster resonance energy transfer
spFRET	single particle Förster resonance energy transfer
EMSA	electrophoretic mobility shift assay
Cy3 and Cy5	fluorescent dyes cyanine 3 and cyanine 5
E_PR_	proximity ratio (FRET efficiency without correction for detection sensitivity and quantum yields of fluorophores)
LN_P, LN_M, LN_D	nucleosomes with one 20 bp linker labeled with Cy3/Cy5 at positions 13/91 bp, 35/112 bp or 57/135 bp, respectively
CN_P, CN_M, CN_D	core nucleosomes labeled with Cy3/Cy5 at positions 13/91 bp, 35/112 bp or 57/135 bp, respectively
