# Supplementary material for: Mechanisms of Nucleosome Reorganization by PARP1
Source: Int J Mol Sci. 2021 Nov 9;22(22):12127. doi: 10.3390/ijms222212127 (PMC8620739; doi:10.3390/ijms222212127)
Supplement: Supplementary file 1 [file ijms-22-12127-s001.zip › ijms-1422570-supplementary.pdf]

Supplementary Figures

Mechanisms of nucleosome reorganization by PARP1

Natalya V. Maluchenko, Dmitry K. Nilov, Sergey V. Pushkarev, Elena Y. Kotova, Nadezhda S. Gerasimova, Mikhail P. Kirpichnikov, Marie-France Langelier, John M. Pascal, Md. Sohail Akhtar, Alexey V. Feofanov, Vasily M. Studitsky

Supplementary Figure S1 PARP1 minimally affects DNA-histone interactions in the nucleosome.

Supplementary Figure S2. Analysis of stoichiometry of PARP1 complexes with core or linker nucleosomes using single particle fluorescence intensity analysis in gel.

Supplementary Figure S3. Typical examples of Epr profiles of CN_P nucleosomes and their complexes with PARP1 described as a superposition of several Gaussians.

Supplementary Figure S4. Analysis of spFRET data for complexes of PARP1 with CN (top) and LN (bottom) nucleosomes.

Supplementary Figure S5. Typical examples of Epr profiles of LN_P nucleosomes and their complexes with PARP1 described as a superposition of several Gaussians.

Supplementary Figure S6. Typical examples of Epr profiles of LN_D nucleosomes and their complexes with PARP1 described as a superposition of several Gaussians.

Supplementary Figure S7. Interaction of PARP1 molecules bound to the ends of nucleosomal DNA.

Supplementary Figure S8. MD equilibration of the free nucleosome model.

Supplementary Figure S9. MD equilibration of the model of nucleosome with one PARP1 molecule (Fig. 5b).

Supplementary Figure S10. MD equilibration of the model of nucleosome with two PARP1 molecules (Fig. 5c).

Supplementary Figure S11. Comparison of equilibrium MD simulations of the free nucleosome model and the model of nucleosome with one PARP1 molecule.

Supplementary Figure S12. Comparison of equilibrium MD simulations of the free nucleosome model and the model of nucleosome with two PARP1 molecules.

Supplementary Figure S13. 12% SDS-PAGE of purified PARP-1 (114 kDa).

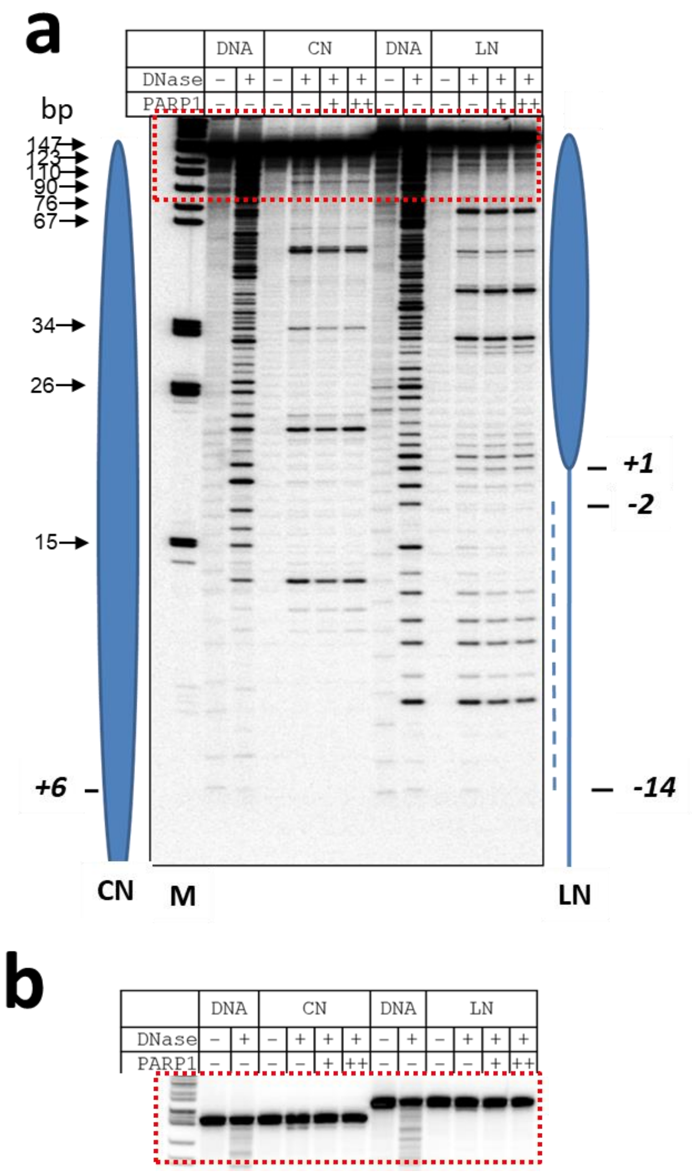

Supplementary Figure S1 PARP1 minimally affects DNA-histone interactions in the nucleosome. (a) analysis of PARP1-nucleosome interactions using DNase I footprinting. End-labeled DNA, CN and LN were incubated in the presence of PARP1 at concentrations that allow binding of either one (+) or several molecules (++) of PARP1 to nucleosomes. DNA was purified and analyzed by denaturing PAGE. The region of the linker DNA partially protected by PARP1 is shown by dashed line. The positions of the nucleosome on the templates are indicated by ovals. Blue line (right part) – a linker region of LN. M – end-labeled pBR322-*Msp*I digest. *Italic* – positions of particular base pairs of the DNA template relative the entrance of DNA into nucleosome. (b) the upper part of the gel shown in panel a (outlined with a red dotted line) that was less contrasted for assessing the loading of the material into the gel.

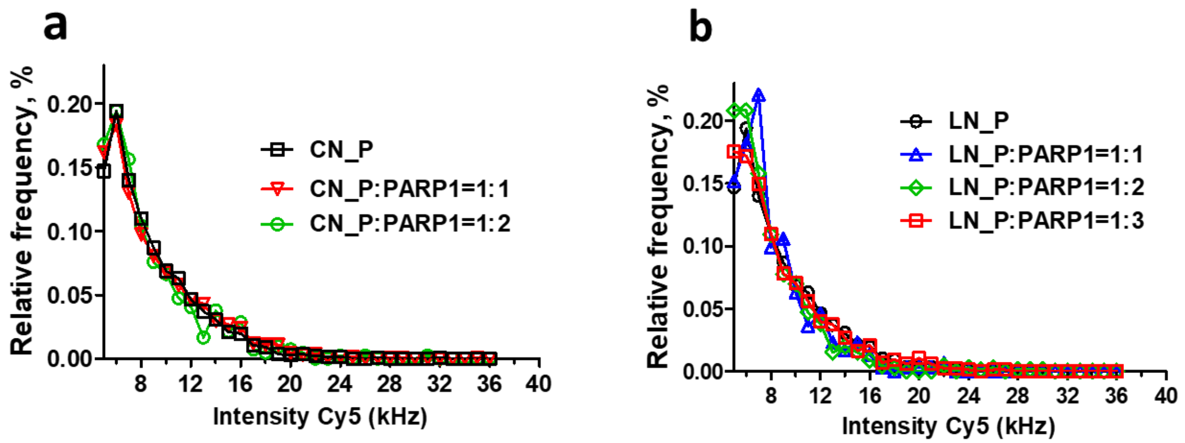

Supplementary Figure S2. Analysis of stoichiometry of PARP1 complexes with core or linker nucleosomes using single particle fluorescence intensity analysis in gel. a. Relative frequency distributions of Cy3 intensities were measured from single nucleosomes CN-P and nucleosome-PARP1 complexes within different bands in the gel like the one shown in Fig. 3a. b. Relative frequency distributions of Cy3 intensities were measured from single LN-P nucleosomes and nucleosome-PARP1 complexes within different bands in the gel like the one shown in Fig. 4a

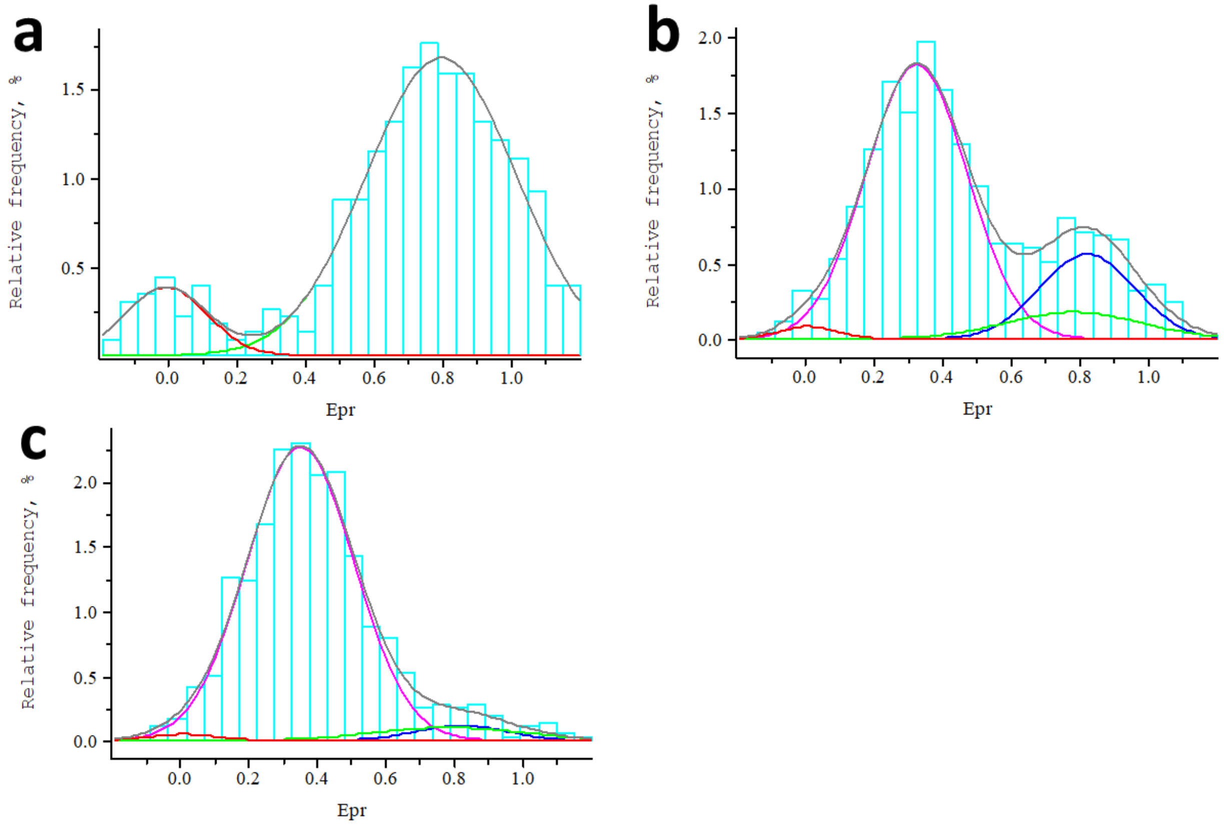

Supplementary Figure S3. Typical examples of Epr profiles of CN_P nucleosomes and their complexes with PARP1 described as a superposition of several Gaussians. The Epr profiles (cyan) of free CN_P nucleosomes (a), and CN_P in the presence of 10 nM (b) or 50 nM (c) PARP1 were fitted as a superposition of HF_1 (green), HF_2 (blue), MF (pink) and LF (red) peaks. The calculated profiles are shown in grey. See text for details.

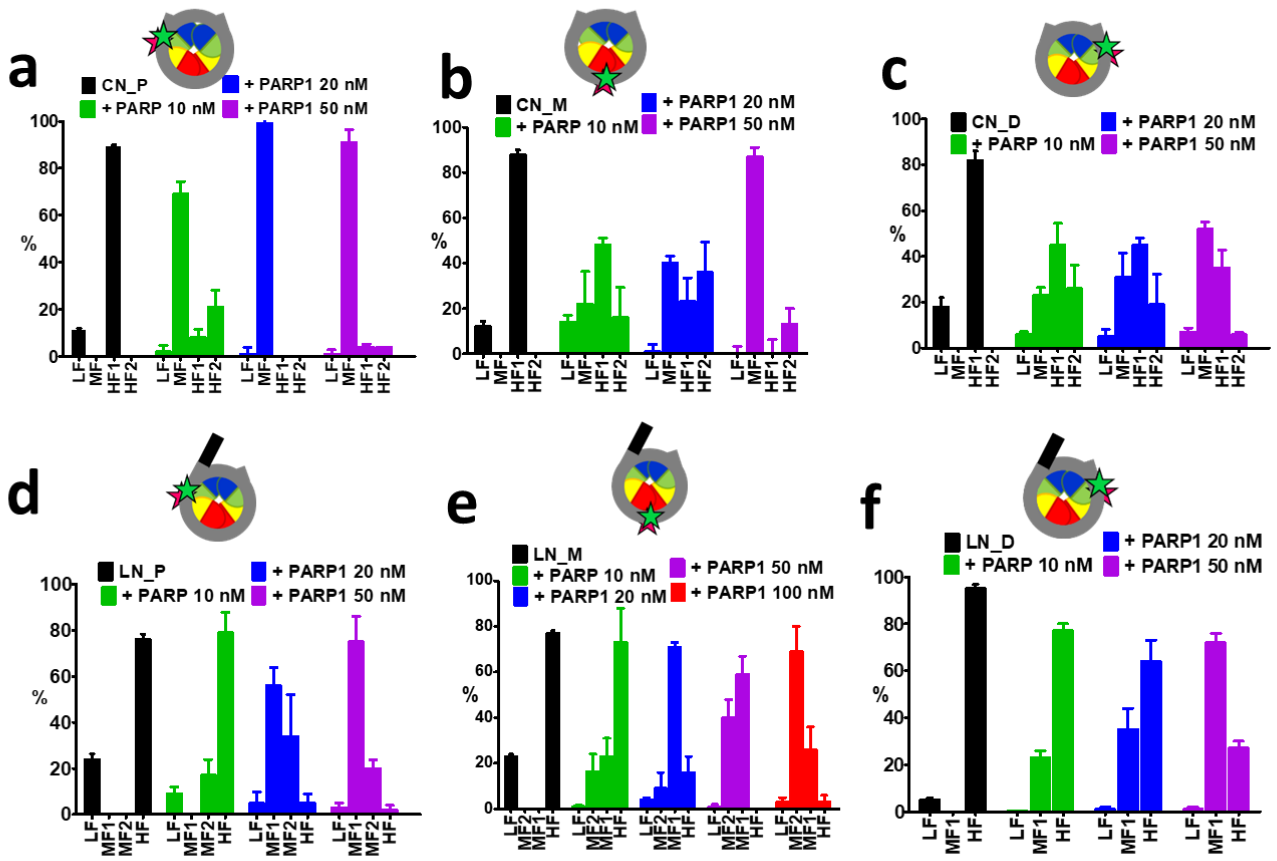

Supplementary Figure S4. Analysis of spFRET data for complexes of PARP1 with CN (top) and LN (bottom) nucleosomes. a-c. The fractions of CN-P (**a**), CN-M (**b**) and CN-D (**c**) nucleosomes forming LF, MF and HF complexes with PARP1 (from the data in **Figure 3b-d**). **d-f.** The fractions of LN-P (**d**), LN-M (**e**) and LN-D (**f**) nucleosomes forming LF, MF and HF complexes with PARP1 (from the data in **Figure 4b-d**).

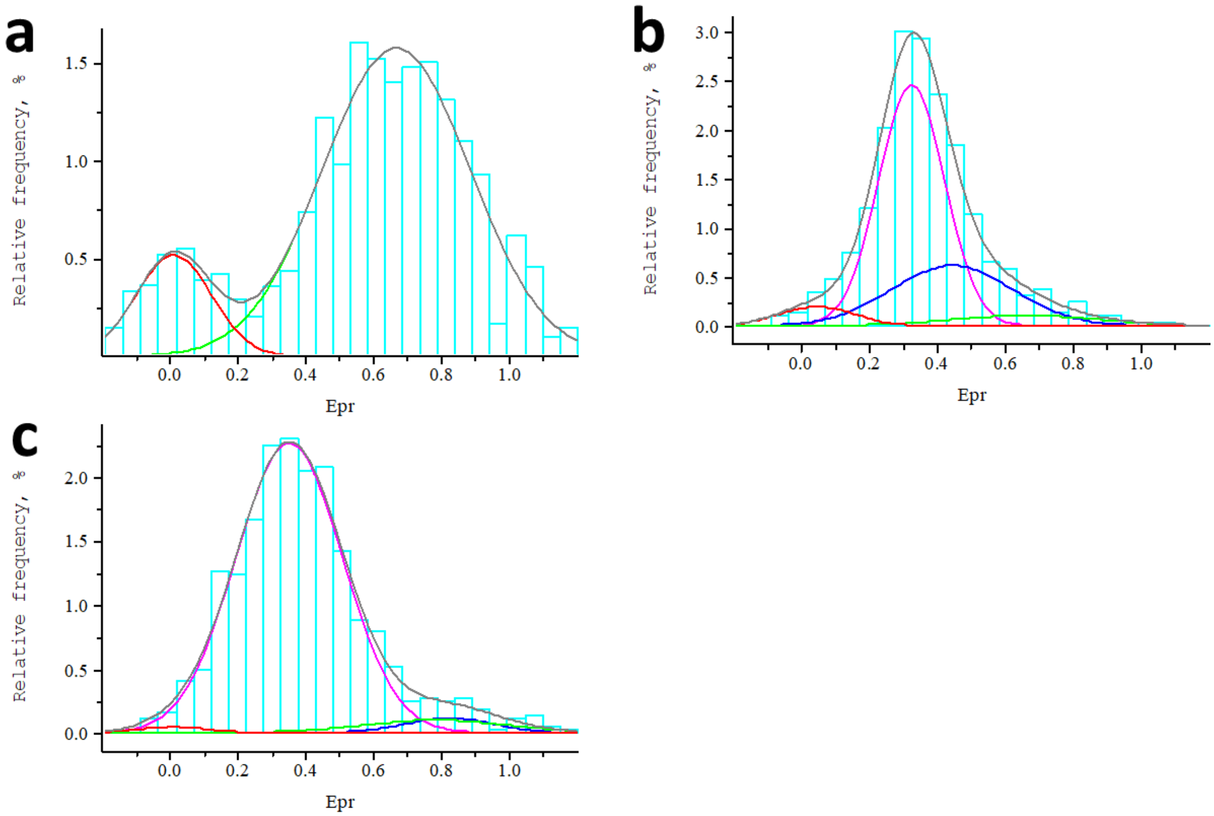

Supplementary Figure S5. Typical examples of Epr profiles of LN_P nucleosomes and their complexes with PARP1 fitted as a superposition of several Gaussians. Designations as in **Supplementary Fig S2.**

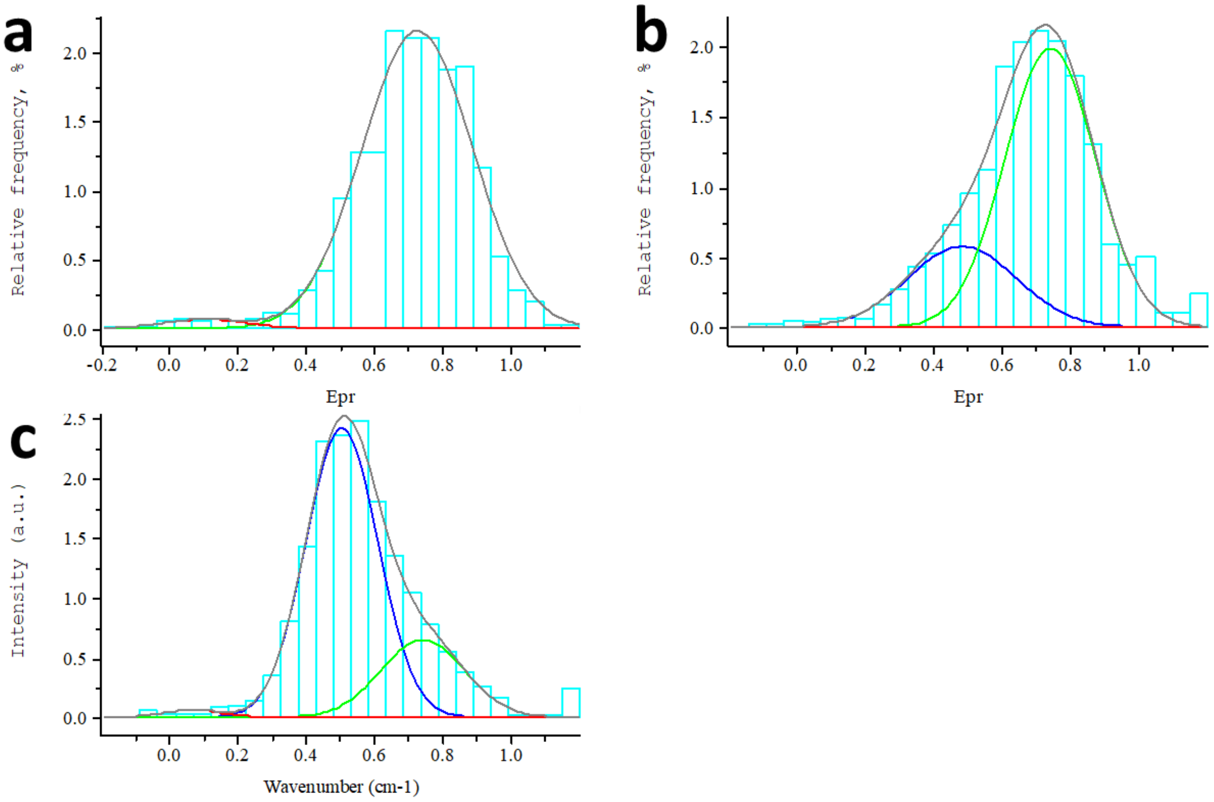

Supplementary Figure S6. Typical examples of Epr profiles of LN_D nucleosomes and their complexes with PARP1 fitted as a superposition of several Gaussians. Designations as in **Supplementary Fig S2.**

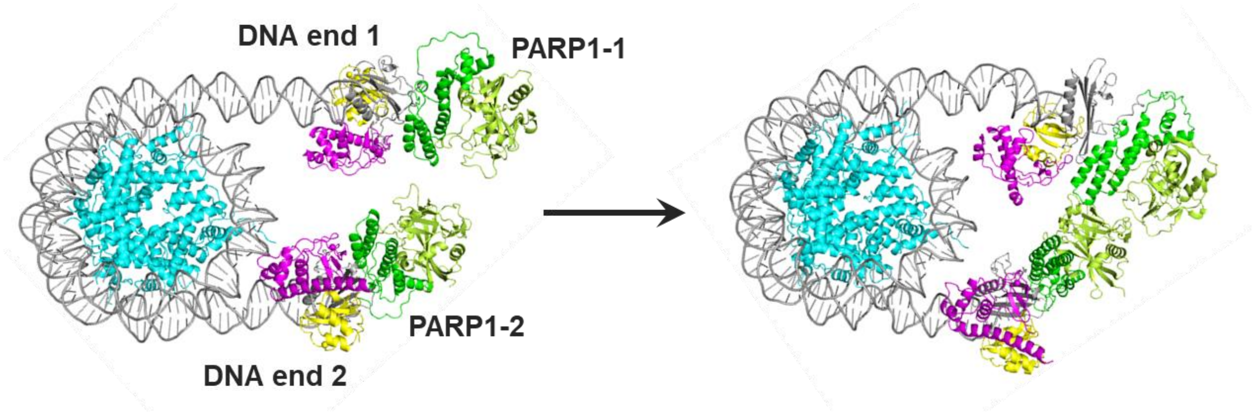

Supplementary Figure S7. Dimerization of PARP1 molecules bound to the ends of nucleosomal DNA. The structures before (on the left) and after MD simulation (on the right) are shown. Designations as in **Figure 5**.

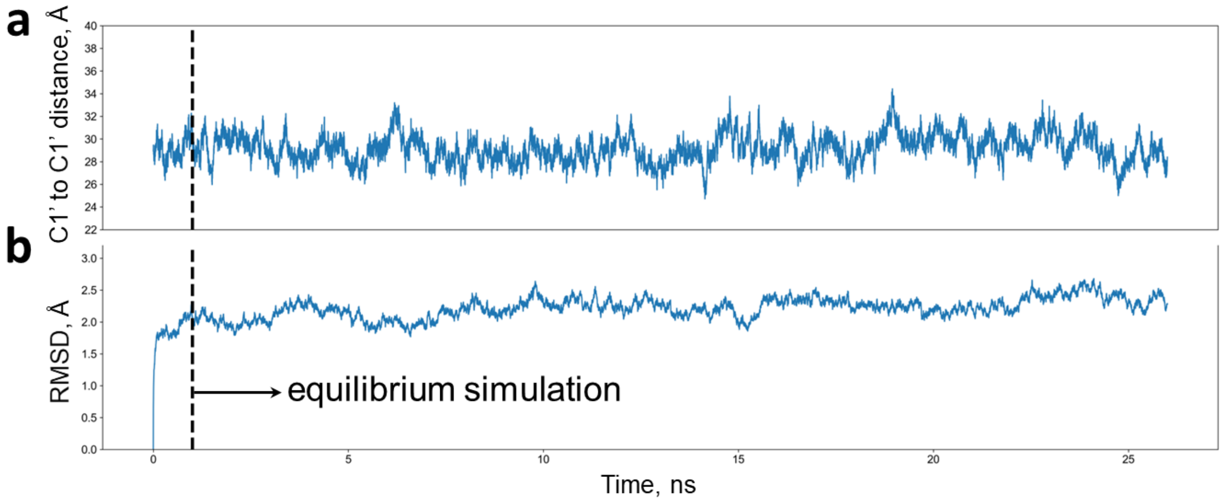

Supplementary Figure S8. MD equilibration of the free nucleosome model (Figure 5a). a. Distance between the C1' atoms of the nucleotides 13 and 91 where the Cy3 and Cy5 labels were attached (see **Figure 1a**). **b.** Root-mean-square deviation (RMSD) of the histone and sugar-phosphate backbone. Trajectory frames were superimposed onto the starting structure by fitting the backbone atoms, and then RMSD of the backbone was calculated. Linker DNA end (20 bp) and terminal bp of the core DNA end were excluded from the RMSD calculation.

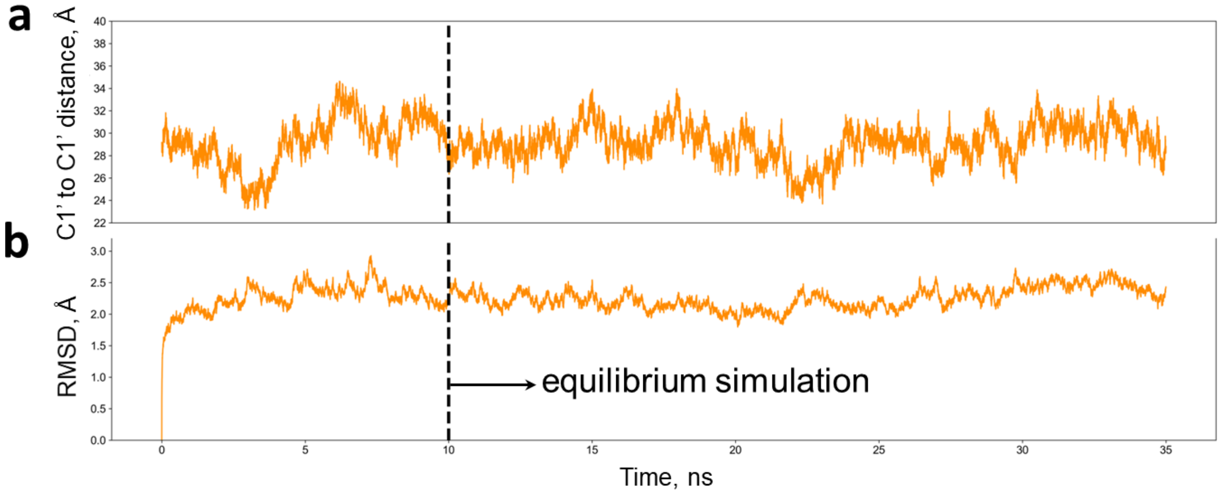

Supplementary Figure S9. MD equilibration of the model of nucleosome with one PARP1 molecule (Figure 5b). a. Distance between the C1' atoms of the nucleotides 13 and 91. (**b**) RMSD of the histone and sugar-phosphate backbone.

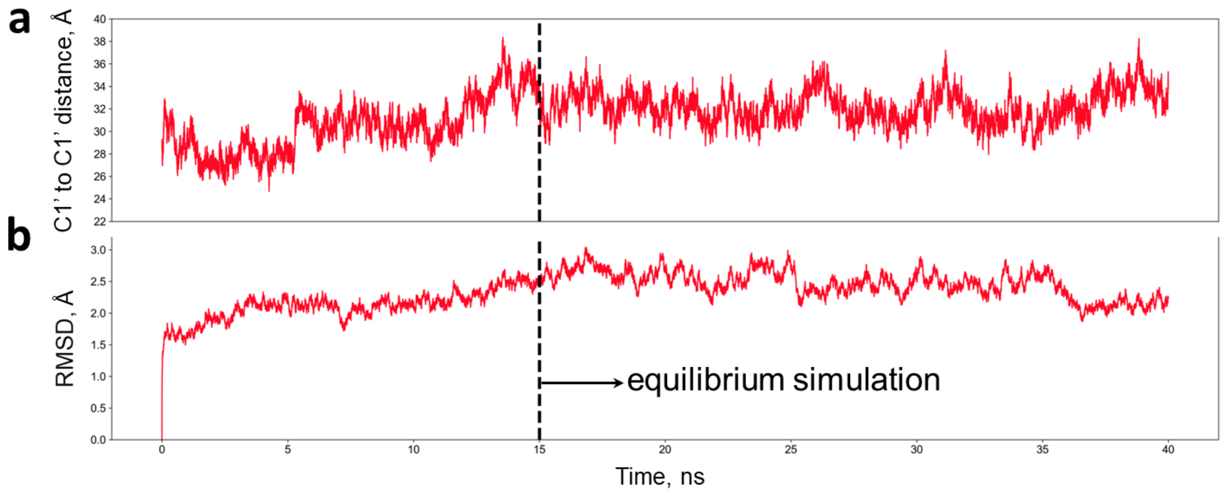

Supplementary Figure S10. MD equilibration of the model of nucleosome with two PARP1 molecules (Figure 5c). a. Distance between the C1' atoms of the nucleotides 13 and 91. **b.** RMSD of the histone and sugar-phosphate backbone.

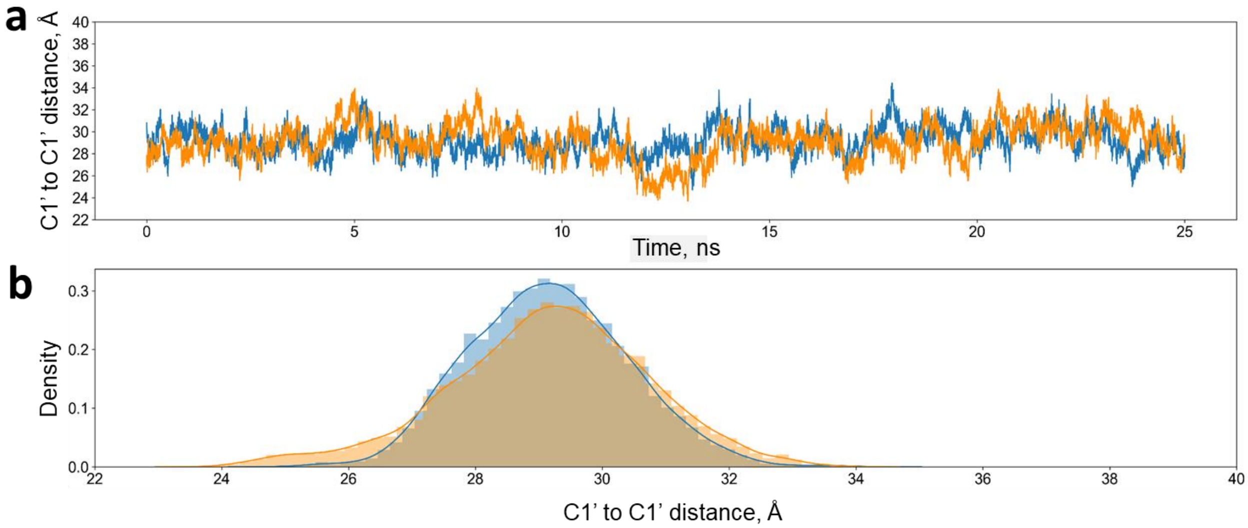

Supplementary Figure S11. Comparison of equilibrium MD simulations of the free nucleosome model (blue) and the model of nucleosome with one PARP1 molecule (orange, Figure 5a and 5b, respectively). a. Distance between the C1' atoms of the nucleotides 13 and 91. **b.** The probability density functions were estimated using kernel density estimation.

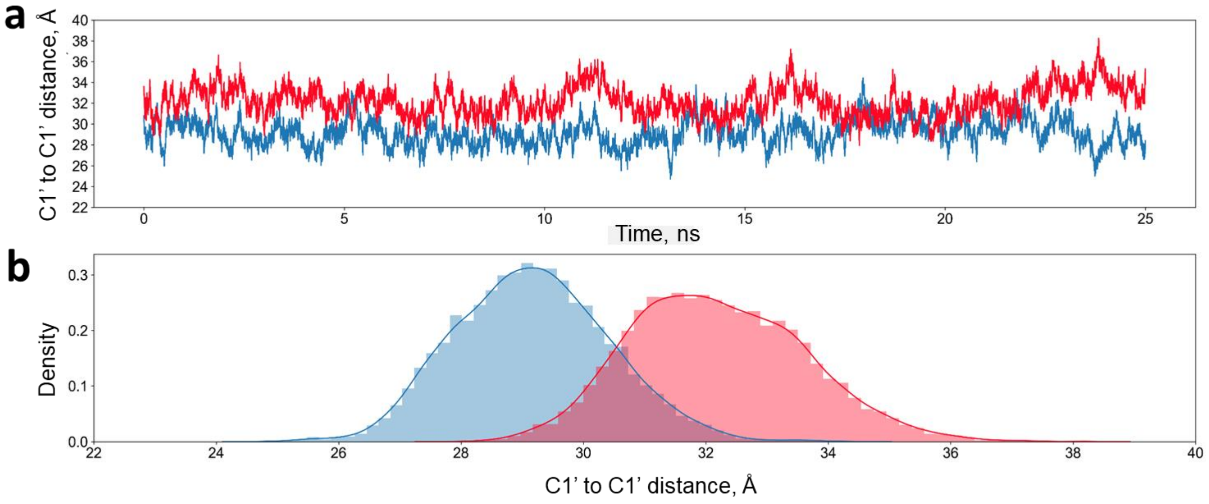

Supplementary Figure S12. Comparison of equilibrium MD simulations of the free nucleosome model (blue) and the model of nucleosome with two PARP1 molecules (pink, Figures 5a and 5c, respectively). a. Distance between the C1' atoms of the nucleotides 13 and 91. **b.** The probability density functions.

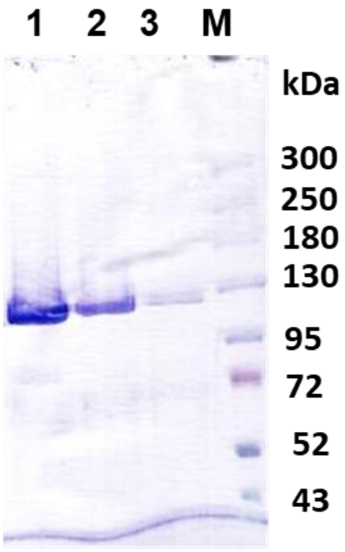

Supplementary Figure S13. 12% SDS-PAGE of purified PARP-1 (114 kDa). PARP-1 electrophoretic mobility on SDS-PAGE was detected by Coomassie staining of polyacrylamide gel. Lines 1,2,3 – three fractions of PARP-1, M - Protein Ladder #SM1851 (ThermoScientific)
